# Pharmacognostical studies of *Hymenodictyon orixence* (Roxb.) Mabb. leaf

**DOI:** 10.4103/0974-7788.64400

**Published:** 2010

**Authors:** Mallesh Reddy, Alka A. Chaturvedi

**Affiliations:** *Department of Botany, Rashtrasanth Santh Tukadoji Maharashtra Nagpur University (RTMNU), Nagpur, India*

**Keywords:** Chromo fingerprinting, pharmacognostical characters, Rubiaceae

## Abstract

*Hymenodictyon orixence* is medicinally important plant found in India, Malaysia and Africa. Due to overexploitation the population of this species has decreased very rapidly. The present study includes pharmacognostical examination of this species. It includes morphological, anatomical, chemical and chromo-fingerprinting characters of *Hymenodictyon orixence* leaf.

## INTRODUCTION

Standardization of natural products is a complex task due to their heterogeneous composition, which is in the form of whole plant, plant part/extracts obtained thereof. To ensure reproducible quality of herbal products, proper identification of starting material is essential.

*Hymenodictyon orixence* is a Rubiaceae member commonly known as '*Bhorsal*' and is mainly known for its wound healing property. It has been reported to have antimicrobial,[1,2] anticoagulant, antiinflammatory and sun screening activity.[3] The present study was carried out to establish methods to facilitate proper identification of *Hymenodictyon orixence* leaf and its powdered form on the basis of morphological, anatomical, chemical and chromo-fingerprinting characters.

## MATERIALS AND METHODS

### Plant material

Leaves of *Hymenodictyon orixence* (Roxb.) Mabb. were collected from Chunala (Manikgad) forest of Chandrapur district (Maharashtra), and properly identified with the help of floras[4-6] at Post Graduate Teaching Department of Botany, Rashtrasanth Santh Tukadoji Maharashtra Nagpur University (RTMNU), Nagpur.

### Pharmacognostical studies

Morphological studies including size, shape, apex, margin, surface and colour were carried out. Other important microscopic characters like epidermal cell number, stomatal index, vein termination, vein islet number and trichomes of both the surfaces was carried out by using standard procedures.[7]

Transverse sections taken by razor were dehydrated, double stained and observed.[8] Some basic chemical reactions were carried with powdered material. Chromo-fingerprints were developed by slight modification of methodology of fluorescence analysis of powdered drugs.[9]

## RESULTS AND DISCUSSION

The leaves of *Hymenodictyon orixence* are simple, opposite decussate, stipulate and petiolate. The leaf measures about 12-30 cm in length and 8-15 cm in width, elliptic in shape, with an abruptly acute apex. The margin is entire, with the base narrowed into a petiole, which is about 2.5–7 cm long and hairy. Dark green above and pale below, the young leaves are silvery and clustered at the end of branches. The stipule is interpetiolar, triangular or broadly ovate, obtuse recurved and deciduous.

**Figure 1:**
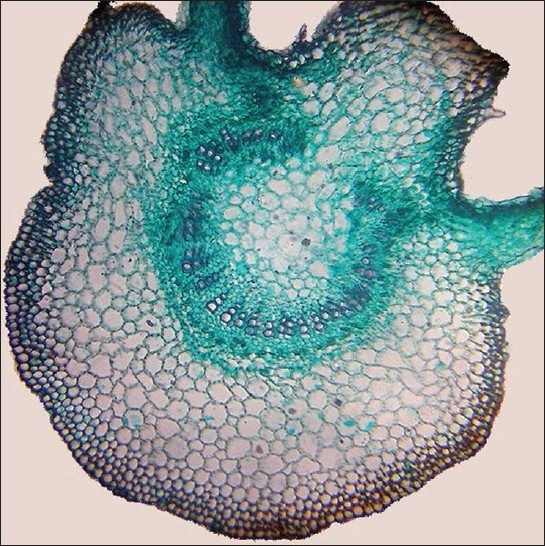
T.S of leaf through midrib

The midrib is elevated on both the surfaces, with the upper being conical and lower semicircular in outline. Inner to the epidermis there is a few layered collenchyma followed by large parenchyma. Vascular bundle shallow is 'U' shaped with incurved margins. The xylem strands are few and embedded in phloem [Figure 1].

**Figure 2:**
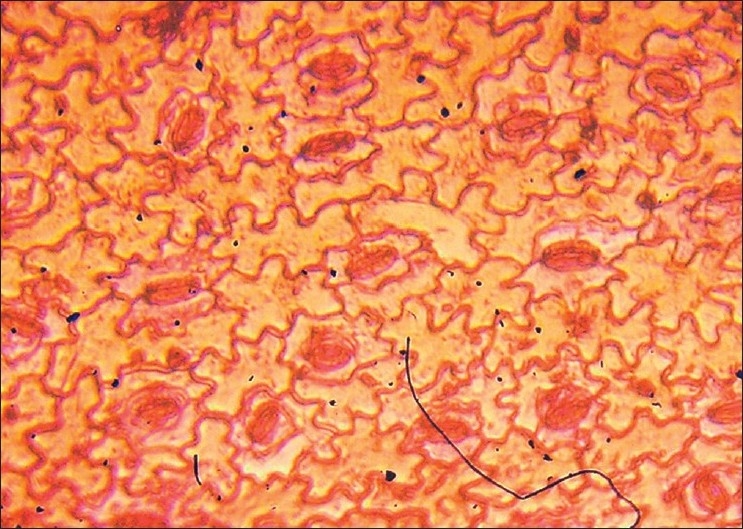
Lower epidermis

**Figure 3:**
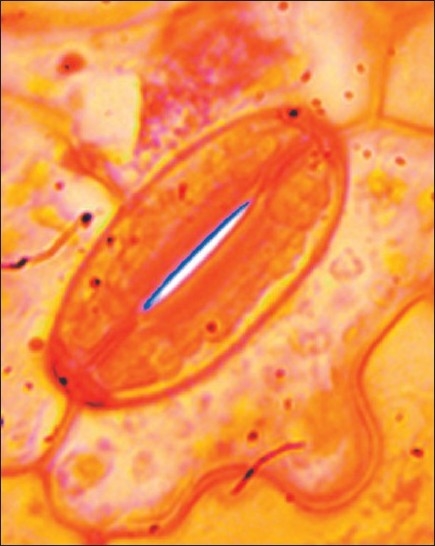
Single stomata

Upper epidermal cells are larger than the lower ones. Both the surfaces have multicellular, uniseriate trichomes measuring up to 250–400 µ, stomata are paracytic [Figures 2 and 3] and restricted to the lower side only. Other important characters are given in the table [Table 1].

**Table 1:** Micro and macroscopic characters

Shape of epidermal cells	Highly irregular
Size of epidermal cells	
Upper surface	2.4 × 10^3^ – 5.8 × 10^3^µ^2^
Lower surface	2.3 × 10^3^ – 3.3 × 10^3^µ^2^
Total number of cells/sq mm	
Upper surface	95°
Lower surface	145°
Stomatal complex length	22–27 µ
Stomatal index	25
Vein termination number	7
Vein islet number	6

Behavior of leaf powder with different chemical reagents was studied to detect the presence of phytoconstituents with colour changes under day light and the results were presented in the table [Table 2].

**Table 2:** Phytochemical tests

Reagent	Colour/precipitate	Constituent
Conc. H_2_SO_4_	Reddish brown	Steroids/ Triterpenoids present
Aq. FeCI_3_	Greenish black	Tannins, flavonoids present
Ammonia solution	No change	Anthracene glycosides absent
Dragendraffs	Orange precipitate	Alkaloids present
Mg–HCl	Red	Fla vonoids present
Alcohol	Mucilaginous precipitate	Gums and mucilage presen
Lead acetate	White precipitate	Tannins present
Libermann- Burchard	Violet	Triterpenoids present
Trim Hill*	Red	Iridoids present

* performed with fresh material

**Figure 4:**
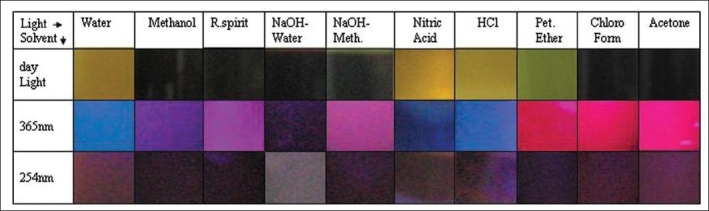
Chromo-fingerprint

The colour of the plant extract is mainly due to its chemical composition. The same extract may appear different in different wavelength of light. Kokashi *et al*,[9] studied the behavior of different vegetable drugs under UV radiation and found that different drugs exhibit different colours and those colours were characteristic for the particular drug. In our study we found a specific colour pattern which is characteristic for *Hymenodictyon orixence*, and hence can be used as a finger print for crude drug identification [Figure 4].
